# Novel Polyethylene Terephthalate Screw Sleeve Implant: Salvage Treatment in a Case of Spine Instability after Vertebroplasty Failure

**DOI:** 10.3390/medicines10010006

**Published:** 2022-12-30

**Authors:** Giacomo Drago, Giulia Pastorello, Paolo Gallinaro, Roberto Zanata, Jacopo Del Verme, Altin Stafa, Enrico Giordan

**Affiliations:** 1 Department of Neuroscience, Università di Padova, 35131 Padova, Italy; 2 Department of Neurosurgery, Aulss 2 Marca Trevigiana, 31100 Treviso, Italy; 3 Department of Neuroradiology, Aulss 2 Marca Trevigiana, 31100 Treviso, Italy

**Keywords:** vertebroplasty, spine, screws, PMMA, complications

## Abstract

Introduction: The management of osteoporotic fractures is sometimes rather challenging for spinal surgeons, and considering the longer life expectancy induced by improved living conditions, their prevalence is expected to increase. At present, the approaches to osteoporotic fractures differ depending on their severity, location, and the patient’s age. State-of-the-art treatments range from vertebroplasty/kyphoplasty to hardware-based spinal stabilization in which screw augmentation with cement is the gold standard. Case presentation: We describe the case of a 74-year-old man with an L5 osteoporotic fracture. The patient underwent a vertebroplasty (VP) procedure, which was complicated by a symptomatic cement leakage in the right L4–L5 neuroforamen. We urgently decompressed the affected pedicle via hemilaminectomy. At that point, the column required stability. The extravasation of cement had ruled out the use of cement-augmented pedicle screws but leaving the pedicular screws alone was not considered sufficient to achieve stability. We decided to cover the screws with a polyethylene terephthalate sleeve (OGmend^®^) to avoid additional cement leakage and to reinforce the screw strength required by the poor bone quality. Conclusion: In the evolving technologies used for spinal surgery, screws sleeve implants such as OGmend^®^ are a useful addition to the surgeon’s armamentarium when an increased pull-out strength is required and other options are not available.

## 1. Introduction

Osteoporosis is a condition that causes the bone to lose structural integrity by decreasing its mineral density and altering the bone formation/resorption mechanism [1], which results in increased susceptibility to fractures [2]. Osteoporosis-related vertebral fractures also occur in the spine and may require cement augmentation using vertebroplasty (VP) or surgical fixation with hardware (i.e., pedicle screws and rods) [2]. As the screw strength in the osteoporotic bone is impaired due to the loss of trabecular bone, several techniques have been developed to improve fixation, such as augmentation with bone cement to increase screw stability in osteoporotic vertebral bodies [1,2]. Furthermore, population aging requires new solutions for increasing a fixation strength that can be applied safely and, if necessary, removed without damaging the surrounding tissue [2].

Cement (polymethyl methacrylate, PMMA) injection procedures, either through VP, kyphoplasty, or augmented screws, are, however, burdened with adverse events—the worst being leakage [3]. In this paper, we present the case of a patient with severe spinal osteoporosis who experienced failure of the L5 VP and developed acute post-procedural L4 radiculopathy and weakness secondary to cement leakage in the right L4–L5 neuroforamen. Our approach to the failure of the VP procedure and neurological status control and fixation will be described in detail in this paper. In this case, the failure of VP required a salvage procedure to control pain and restore neurological function while ensuring stability without using cement augmentation. Therefore, because of the impaired bone quality and the fear of new cement extravasation, we deployed a recently designed braided polyethylene terephthalate screw sleeve (OGMend^®^) to improve the screw pull-out strength. Such a device has rarely been utilized, and large cohort studies on its use are still unavailable in the literature. This case, for the first time to our knowledge, aims to sensitize spine surgeons to the use of additional technologies that can be helpful in select cases in which traditional therapeutic approaches are lacking in efficacy or are not suitable.

This case report conforms to the CARE guidelines (for CAse REports).

## 2. Case Report

Institutional review board approval was not required for this case report, as per our institution’s policy. Moreover, data from this case were anonymized, and sensitive pieces of information were deleted from the images. The patient provided informed consent and agreed to publish anonymized personal data.

**Figure 1:**
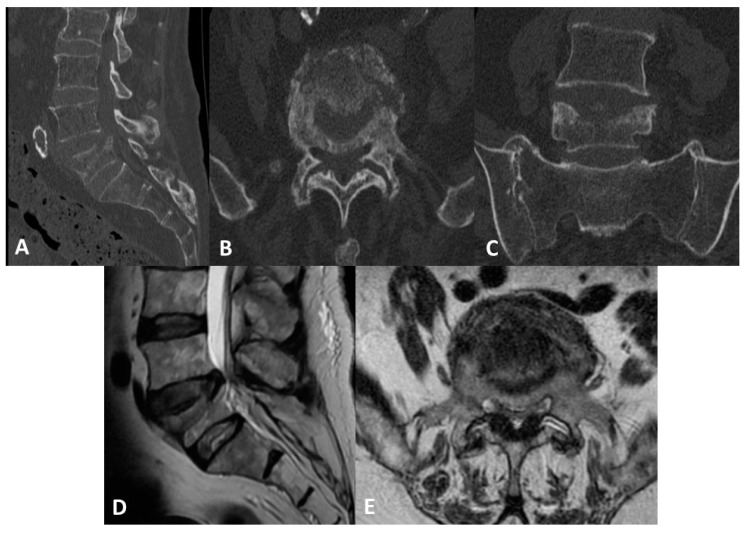
(**A**) Sagittal CT scan of the L5 fracture; (**B**) axial CT scan at the fracture level; (**C**) coronal CT scan; (**D**) sagittal MRI scan of the L5 fracture; (**E**) axial MRI scan at the fracture level.

**Figure 2:**
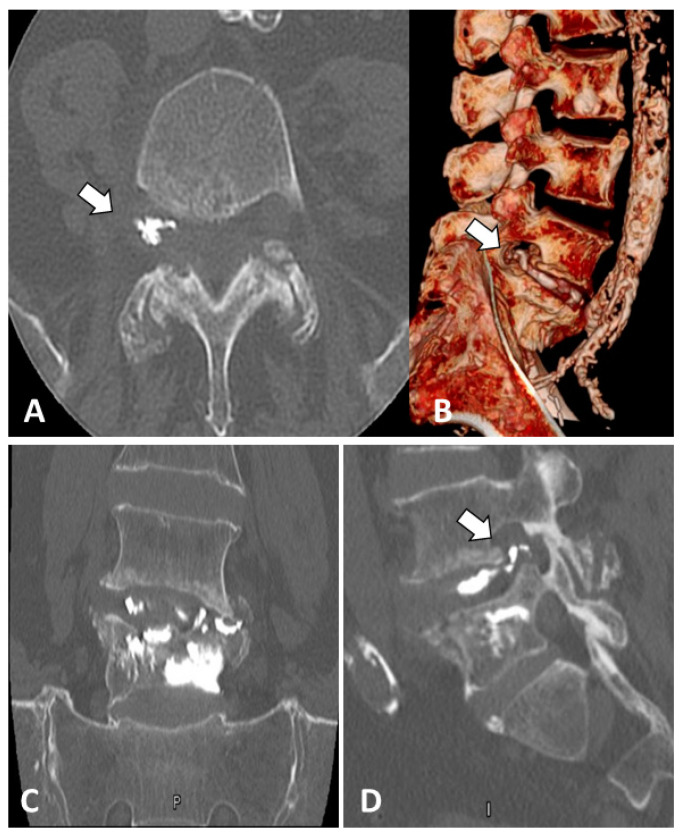
(**A**) Axial CT scan at the fracture level, documenting cement leakage in the neuroforamen (white arrow); (**B**) A 3D CT reconstruction of the lumbar spine: cement extravasation visible in the right neuroforamen (white arrow); (**C**) sagittal CT scan of the L5 fracture after VP; (**D**) sagittal CT scan at the fracture level, documenting cement leakage in the neuroforamen (white arrow).

A 74-year-old male with a medical history of hypertension, cardiac disease, prostate cancer, and obstructive pulmonary disease was referred to us to evaluate his acute back pain. A lumbar CT scan showed an L5 fracture (Figure 1) in what we consider, based on CT examination (i.e., the appearance of diminished bone density as well as cortical thickness and loss of bony trabeculae, etc.), a severely osteoporotic spine. We did not perform a specific examination to assess the suspected osteoporosis degree, and we only performed an MRI scan to exclude other causes of fracture (i.e., neoplasms or infection). Moreso, the average Hounsfield Units (HU) value for the L5 vertebral measured on the CT scan was 47 units, which is under the HU cutoff value considered highly predictive for osteoporosis (the average HU for the selected vertebra was calculated on an oval region of interest placed over an axial image of the L5 mid-body. PACS software automatically calculates the average CT HU for the selected region of interest. Reported values in the recent literature are 54.7 ± 25.2 HU units for osteoporotic bone and 120.8 ± 41.8 HU for normal bone [4]. At that point, we opted for conservative treatment with a lumbar orthosis. At 30 days, the patient’s lower back pain had worsened, and another CT scan showed a progression in the L5 fracture. The patient was counseled to perform dual-energy X-ray absorptiometry, confirming the diagnosis of osteoporosis (T-score: −2–4) and undergoing endocrinology assessment and adequate therapy. The patient was a proposed L5 vertebroplasty, but the percutaneous procedure was complicated by cement extravasation through a lateral fracture cleft (Figure 2). The cement reached the anterior-inferior portion of the L4–L5 right neuroforamen, causing the patient severe L4 radiculopathy with decreased strength in the right quadriceps femoris (3/5 on the Medical Research Council scale). In our opinion, these adverse events discouraged any other type of cement-related procedures, even in neighboring vertebras, due to the risk of additional cement extravasation.

**Figure 3:**
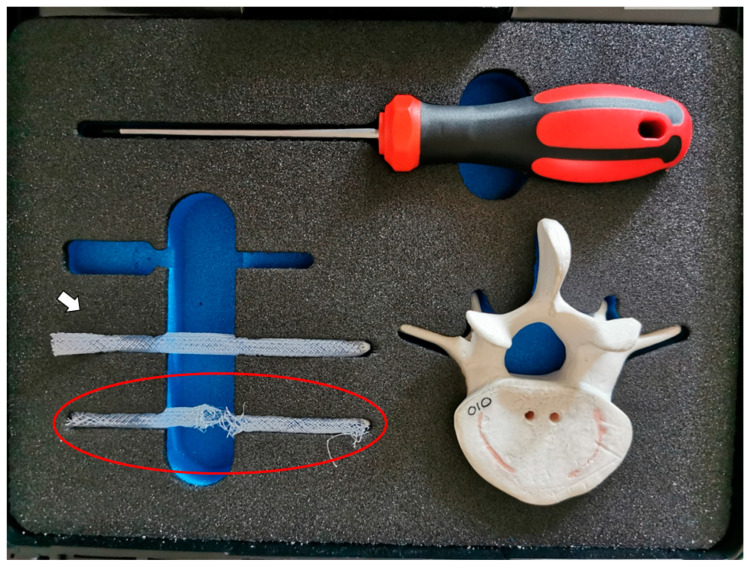
An example of an OGmend^®^ kit with an intact sleeve implant (white arrow) and a de-interlaced one (red circles) showing the net of interwoven polyethylene terephthalate fibers.

To control the pain and restore neurological function, the patient received an open L4–S1 instrumentation with pedicle screws and rods (Stryker Corp., Kalamazoo, MI 49002 USA) and decompression via an L4 hemilaminectomy and medial facetectomy. The L5 vertebral body, because of the presence of cement, was not available for screw placement, and thus a bridging fixation from L4 to S1 was considered. Unfortunately, in the S1, we could not insert screws in a bicortical manner, which could have helped increase the pull-out strength. In order to enhance the quality of the screw-to-bone interface, the pedicle screws at L4 and S1 were covered with an OGmend^®^ implant (Woven Orthopedic Technologies, LLC. Manchester, USA), a polyethylene terephthalate sleeve (Figure 3). This was done to prevent additional cement leakage and reinforce the screw’s strength, considering the poor bone quality.

**Figure 4:**
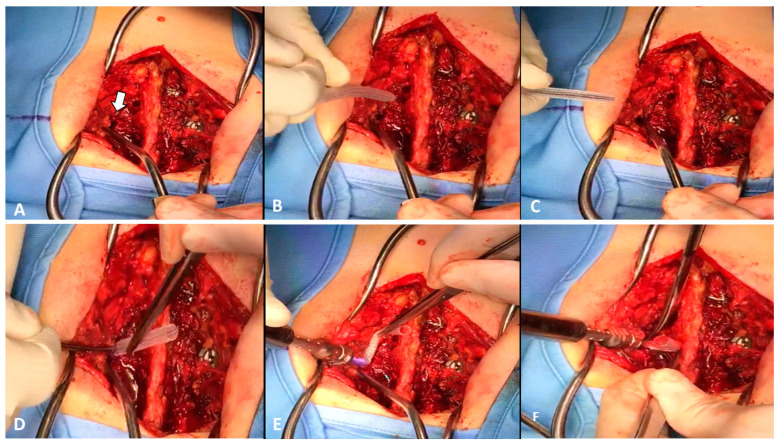
Intraoperative imaging. (**A**) Pedicle hole for screw insertion (white arrow); (**B**) The sleeve over the pedicle probe; (**C**) The pedicle probe, inserted through the hole, to help pedicle insertion; (**D**) pushing the sleeve inside the pedicle hole with the pedicle probe; (**E**) Screw placement inside the OGmend^®^ sleeve; (**F**) After complete insertion the sleeve excess is cut with scissors.

After selecting the appropriate screw length, we partially cut the sleeve with scissors. We left 3–5 fibers attached between the end of the cut and the rest of the implant to create a tab that could be held for proper placement and positioning during the insertion (Figure 4). At that point, the inserter was passed through the center of the sleeve, bypassing the tab and whole length of the sleeve We placed OGmend^®^ inside the hole created by the pedicle tap, leaving the remaining portion slightly above the hole level. We used a tab to reposition the OGmend^®^ by pulling the tab proximally (Figure 4). While holding the tab, we inserted the screw into the OGmend^®^ implant. We drove the screw into the hole until approximately 50% of its length, cut the remaining part, and completed the insertion.

**Figure 5:**
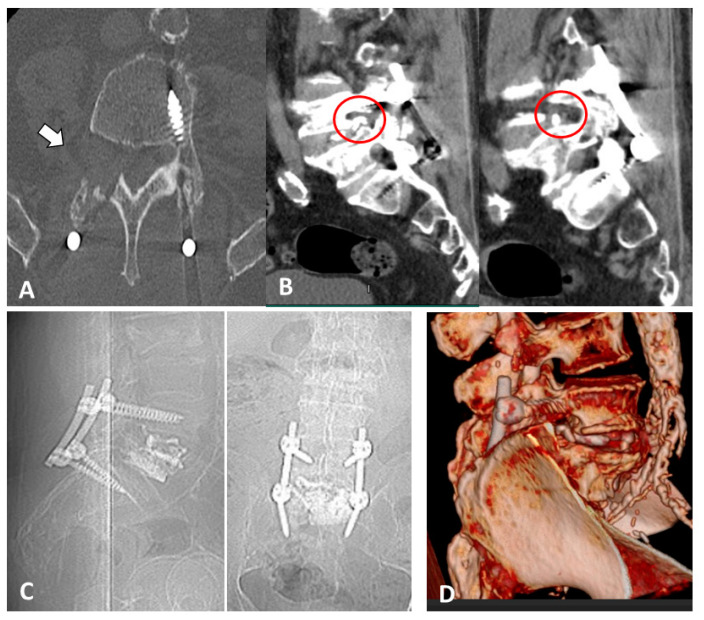
The 3-month follow-up, post-operative imaging. (**A**) Axial CT scan at the L4–L5 right neuroforamen, showing the foramen enlargement after hemilaminectomy and foraminotomy (white arrow); (**B**) sagittal CT scans showing neuroforamen enlargement over cement leakage (red circle); (**C**) sagittal (left) and coronal (right) post-operative X-rays; (**D**) 3D CT reconstruction of the lumbar spine: foramen enlargement over cement leakage.

The procedure was well tolerated and had no immediate post-operative complications. At 3 months, the patient was pain-free, and the incisions were healed with no evidence of prominent instrumentation. The radiographs showed that the instrumentation was intact and well-aligned (Figure 5).

**Figure 6:**
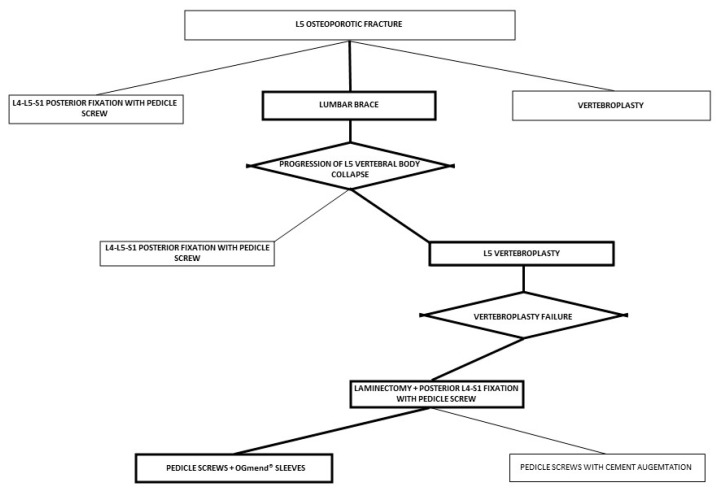
Decisional approach summary. The bold black line highlights the decisional approach chosen compared to the thin black line, which shows the alternative strategies that were not taken.

A graphical summary of our decisional process is pictured in Figure 6.

## 3. Discussion

Injected cement may leak into different anatomical compartments, including the prevertebral soft tissue (6–52.5% of cases), the spinal canal (1.2–37.5%), intervertebral disk (5–25%), prevertebral veins (5–16.6%), and epidural veins (16.5%) [3]. Catastrophic cases of leakage in the inferior vena cava and lungs have also been reported [5]. The cement extravasation rates reported in the literature are 11–76% for vertebroplasty and 4.8–39% for kyphoplasty [5,6,7]. Compression spine fractures imply a disruption of venous drainage; therefore, cement extravasation during VP or kyphoplasty is less likely [8].

Despite the rates reported in the case series, in cadaveric studies, 100% of the patients showed some degree of extravasation, and 92% of injected VBS had CT evidence of cement leakage. Almost one-third of the patients in these studies were found to have one or more clinically worrisome extravasation events, while in most cases, the extravasation was only a small (1 mm) protrusion of cement outside the external osseous borders of the vertebral body. These studies show that the frequency of leakage was significantly higher than suggested by fluoroscopy during the augmentation procedures and that surgeons always believed they had stopped the injection before any cement extravasation [9].

Cement viscosity, bone density, cement injection rate and volume, presence, or absence of vertebral body fracture, and the level of cement injection can influence the risk of cement extravasation [7], and surgeons must act on them to decrease the risk. Additionally, a significant parameter to consider is the timing of the cement injection concerning the fracture. Several studies highlighted how the best candidate for percutaneous vertebroplasty are patients with subacute fractures, between 1 to 3 months, in which satisfactory results can be achieved with a lower risk of cement leakage than that of acute fracture patients (<1 month). It may be possible that, in our case, the fracture was still in his acute phase (30 days) and thus possibly leading to an increased risk of cement extravasation [4]. However, we believe that, eventually, the experience and skills of the physicians performing the procedure are the paramount factors for success.

Since 2007, only a few cases of cement leakage after VP with symptomatic lesions have been reported in the literature [10,11,12]. In these occurrences, the hard cement compressed, heated up, and even adhered to the dura, thus producing neurological symptoms [13]. In some cases, neurological deficits were also associated with persistent spinal instability. In these instances, the most common salvage procedure is the laminectomy for urgent spinal cord decompression and, when required, spinal stabilization. In a very similar case of cement leakage anatomical position, Wagner et al. recently presented a minimally invasive transforaminal endoscopic solution for VP and kyphoplasty cement leakage, and their patients’ pain improved immediately after surgery [13,14]. In addition, Senturk et al. recently described a minimally invasive endoscopic translaminar technique for removing cement leakage fragments after PVP [10,15,16].

In our case, it was paramount to decompress the neurological structures while also stabilizing the spine. Unfortunately, osteoporosis decreases the fixation strength of pedicle screws by 40% to 80% [17]. Further, augmentation of pedicle screws in an osteoporotic bone is one of the most commonly used methods of osteoporotic spine fixation or revision surgery. However, even here, concern exists that cement-augmented pedicle screws may fail by posterior displacement while still bound to cement [17] and that with fenestrated screws, there may be a risk of cement leakage and neurological complications, especially if the screw is too short and its fenestrated portion is close to the foramen [17].

In our case, the pull-out strength was compromised by osteoporosis, and cement augmentation could not be used, in our opinion, for the risk of further extravasation. Therefore, we adopted a screw implant system. Different strategies, i.e., mechanical devices such as intrapedicular bone anchors, are being tested to improve screw pull-out strength [2]. However, such devices are quite expensive and require storage space and specific training in order to implant them correctly. Conversely, the OGmend^®^ implant is simply a sleeve that acts as a bone plug-in before inserting the pedicle screw. Additionally, it is compatible with screws that already exist and does not occupy storage space. Such advancements in material and implants in the field of osteoporosis are mandatory to overcome the limitations of standard treatments (i.e., VP or pedicle screw with cement augmentation) [18,19]. Further, new therapeutic strategies are mandatory in medical and surgical fields.

We believe that shifting to a less aggressive surgical approach by progressively integrating the screw into the bone instead of anchoring them with cement may benefit the biomechanical aspect of the construct and neighboring vertebra [19,20]. The implant is made of thermoplastic polymer resin, used in synthetic fibers (most notably, dacron sutures) and is biocompatible. The FDA approved its use over 40 years ago. The sleeve can be used on common commercial pedicle screws of different sizes. It offers a scaffold to enhance the bone–screw interface and favors bone in-growth due to its porosity and bone remodeling over the screw, thus reaching a pull-out strength comparable to that achieved in healthy bone. The integration aspect is paramount, as it offers the possibility of acting on the bone’s natural ability to heal the fracture without impairing it with cement.

In addition, animal or human studies have demonstrated the safety of this device [21]. Two studies were conducted on ovine animal models. The first one evaluated the efficacy of the screw retention technology, and the second one simulated screw loosening and showed the biomechanical and histological results of achieved stabilization and fusion, demonstrating the efficacy of this solution on improved screw retention. Thus, this ancillary device could also be used in case of screw loosening or on a routine basis when the surgeon feels the screw construct needs to be strengthened.

The main limitation of this case is the inherent low level of evidence (level IV), which limits the generalizability of our conclusions. Unfortunately, the literature on these devices is still scarce and mostly based on animal models for OGmend’s safety and efficacy. Large cohort studies, as well as long-term histological specimens for assessing osteointegration, are expected to confirm the features of the screw sleeve in terms of both osteointegration and safety.

## 4. Conclusions

In the evolving technologies used for spine surgery, screw augmentation implants such as OGmend^®^ are a useful addition to the surgeon’s armamentarium for salvage procedures or routine screw placement when an increased pull-out strength is required, such as in cases of osteoporosis. A spinal surgeon should bear in mind that innovative options to help them adapt to each case’s requirements are available.

## Data Availability

No new data were created or analyzed in this study. Data sharing is not applicable to this article.
